# Antimicrobial resistance and pathogen distribution in hospitalized burn patients: A multicenter study in Southeast China

**DOI:** 10.1097/MD.0000000000011977

**Published:** 2018-08-24

**Authors:** Lin Li, Jia-xi Dai, Le Xu, Zhao-hong Chen, Xiao-yi Li, Min Liu, Yu-qing Wen, Xiao-dong Chen

**Affiliations:** a Department of Burns, Fujian Medical University Union Hospital, Fuzhou; b Department of Nursing, Xiamen Medical College, Xiamen; c Department of Nursing, Fujian Medical University Union Hospital, Fuzhou; d Department of Burns, The 180th Hospital of Chinese People's Liberation Army (PLA), Quanzhou; e Department of Burns, The 92nd Hospital of Chinese People's Liberation Army (PLA), Nanping; f Department of Burns, The First Hospital of Longyan City, Longyan, Fujian Province, China.

**Keywords:** bacteria, burns, drug resistance

## Abstract

Burn infections pose a serious obstacle to recovery. To investigate and analyze the antimicrobial resistance and distribution of pathogenic bacteria among hospitalized burn patients. A 3-year retrospective study was conducted in the southeast of China.

The electronic medical records system was used to collect all clinical data on 1449 hospitalized patients from Fujian Medical University Union Hospital, the 180th Hospital of Chinese People's Liberation Army (PLA), the 92nd Hospital of PLA, and the First Hospital of Longyan City.

A total of 1891 strains of pathogenic bacteria were detected from 3835 clinical specimens, and the total detection rate was 49.3% (1891/3835). The main pathogens were gram-negative bacteria (1089 strains; 57.6%), followed by gram-positive bacteria (689 strains; 36.4%), and fungi (113 strains; 6.0%). The predominant five bacteria were *Staphylococcus aureus* (19.0%), *Acinetobacter baumannii* (17.6%), *Pseudomonas aeruginosa* (16.7%), *Klebsiella pneumoniae* (7.4%), and *Enterococcus faecalis* (4.5%). Methicillin-resistant *Staphylococcus aureus* (MRSA) accounted for 74.1% (265/359) of *S aureus* isolates. *Staphylococcus epidermidis* accounted for 40.6% (69/170) of coagulase-negative staphylococcal isolates, 72.5% (50/69) of which were methicillin-resistant *Staphylococcus epidermidis* (MRSE). Both MRSA and MRSE were 100% resistant to penicillin and ampicillin. *A baumannii* was the most commonly isolated strain of gram-negative bacteria with 100% resistance to ampicillin, amoxicillin, amoxicillin/clavulanic acid, and aztreonam. More than 80% of *K pneumoniae* isolates were resistant to ampicillin, amoxicillin and cefazolin. More than 80% of *Escherichia coli* isolates were resistant to ampicillin, piperacillin, cefazolin, amoxicillin, tetracycline, and sulfamethoxazole trimethoprim. The detection rates of extended-spectrum β-lactamases (ESBL) among *K pneumoniae* and *E coli* isolates were 44.6% (62/139) and 67.2% (41/61), respectively. Low-resistance antibiotics included teicoplanin, tigecycline, vancomycin, and linezolid.

The pathogens presented high resistance to antimicrobial agents, especially MRSA and *A baumannii*. Monitoring of bacterial population dynamics should be established to inhibit the progression of bacterial resistance.

## 1 Introduction

Burn infections are a serious hindrance to patient recovery. Infections have been estimated to account for 75% of burn patient deaths.^[1,2]^ The damage of protective skin barrier and the damage of humoral and cellular immunity accelerate the colonization of skin microorganism.^[3]^ In addition, the gastrointestinal tract bacterial translocation and invasive diagnosis and treatment procedures, such as tracheal intubation, invasive central veins or ductus arteriosus, and catheterization, also contribute to the incidence of infection.^[4]^ Moreover, a serious problem in China is antibiotic overuse, which promotes the emergence of antimicrobial-resistant pathogens.

By reviewing the variable history of the burn wound bacterial ecology, we have observed changes with time^[5]^ and climate.^[6]^ Within the same hospital moreover, bacterial drug resistance varies in response to local or systemic medications.^[7]^ Therefore, the timely and pre-emptive understanding of the bacterial epidemiologic distribution and antimicrobial-resistance patterns among burn patients is of critical importance.

In this study, a retrospective analysis was conducted concerning the pathogen distribution and antimicrobial resistance of a total of 1891 isolates from 1449 patients with nosocomial infections in 4 burn wards in Fujian province (located in the southeast of China) from January 2013 to December 2015. This study could serve as a reference for the prevention or treatment of burn infections and the rational use of antimicrobials.

## 2 Materials and methods

### 2.1 Patients

Patients treated in the burn wards of Fujian Medical University Union Hospital, the 180th Hospital of PLA, the 92nd Hospital of PLA, and the First Hospital of Longyan City between January 2013 and December 2015 were included in this retrospective analysis. Patients with incomplete data will be excluded. Fujian Medical University Union Hospital is a Class A tertiary provincial public hospital. Its burn department, established in 1976, is the burn center for Fujian province. The center has 3 subdivisions, including burn treatment, plastic surgery, and rehabilitation. The center consists of an independent outpatient department for wound treatment, independent operating rooms, a burn intensive care unit (BICU), and 82 ward beds, which include 8 sickbeds in the BICU and 6 for emergencies. Between 2013 and 2015, a total of 2552 new burn patients were admitted to this center. The 180th Hospital of Chinese People's Liberation Army (PLA), a Class A tertiary military hospital with 79 beds in its burn department including 7 sickbeds in the BICU, treated 4591 burn patients between 2013 and 2015. The 92nd Hospital of PLA is also a Class A tertiary military hospital with a total of 44 beds in its burn department, which treated 956 burn patients during the same period. The First Hospital of Longyan City is a Class A tertiary municipal public hospital and the only center of its type in the western Fujian province. Its burn department is equipped with 20 sickbeds and treated 727 new burn patients in 2013 to 2015. The condition and treatment of burn patients at these 4 burn wards are satisfactorily representative of the entire Fujian province.

The inclusion criteria were as follows: burn data from the first admission were extracted using the International Classification of Diseases, Tenth Revision (ICD-10) codes in the X00 to X19 range; patients admitted to these 4 hospitals for >24 hours or who died after arrival at the hospital were studied. The following categories of patients were excluded: readmissions for scar contracture; outpatients; inpatients not diagnosed with a burn as the primary cause of admission; and incomplete clinical data.

A total of 1449 patients were enrolled in our study (843 cases, 160 cases, 160 cases, and 286 cases, respectively, from the aforementioned 4 burn departments). The patients’ ages ranged from 8 days to 94 years (median age, 32 years; interquartile range, 29 months to 48 years). The subjects included 996 men and 453 women. The total burn surface area (TBSA) ranged from 1% to 99% (median TBSA, 11%; interquartile range, 4%–24%). The burn depths were between II and III with 446 cases of flame burns, 732 cases of scalding, 108 cases of contact burns, 105 cases of electrical burns, 34 cases of chemical burns, and 24 cases of other burn etiologies. Mild burns accounted for 313 cases, whereas 709 cases were moderate burns, 216 cases were severe burns, and 211 cases were extremely severe burns.

In total, 1891 strains of pathogenic bacteria were cultured from 3835 clinical specimens obtained from and distributed as follows: wound secretions, 1992; blood samples, 979; respiratory secretions, 595; central venous catheter specimens, 133; and other sources (such as urine, stool, and tissue fluid), 136.

The ethics committee of Fujian Medical University and each collaborating institution reviewed and approved the study protocol. All collaborating institution provided written consent for their information to be collected and used for research, and they had the right to withdraw from the study at any time without prejudice.

### 2.2 Sample collection

Wound secretions were collected at patient admission and during hospitalization at least once; the required volume for each sample was >1 mL. Sputum secretions were collected from patients receiving preventive tracheotomy or ventilator support; the required sample volume for each was >2 mL. Central venous catheter samples were obtained at catheter replacement; the minimal required sample volume was 5 cm. Blood samples were obtained when the patient's temperature rose >38.5 °C or was <36 °C (sourced from 2 peripheral blood collection sites or 1 peripheral blood and 1 intraductal blood site using 2 sets of 4 bottles with aerobic and anaerobic cultures obtained for each sample; the required volume for each sample was 5–10 mL for adults and 2–5 mL for children). Urine samples were obtained when patients had irritative urinary tract symptoms (using 1 set of 2 bottles with aerobic and anaerobic cultures obtained for each sample; the required volume for each sample was 5–10 mL). Stool samples were obtained when patients had diarrhea (using 1 set of 2 bottles with aerobic and anaerobic cultures obtained for each sample; the required volume for each sample was at least 1 mL). For patients with suspected sepsis, all aforementioned sample cultures were obtained for 3 consecutive days. Patients were treated based on the revised guideline, Diagnostic Criteria and Treatment Guideline for Infection of Burns and Guideline for Diagnosis, Prevention and Treatment of Invasive Fungal Infection after Burn Injury.^[8]^

### 2.3 Species identification and antibiotic sensitivity

Species identification and antimicrobial sensitivities were assessed by the laboratory staff of the 4 hospitals using the Kirby-Bauer (K-B) disk diffusion method employing drug-containing test disks, culture medium and quality control strains (*Staphylococcus aureus* ATCC 25923, *Pseudomonas aeruginosa* ATCC 27853, *Escherichia coli* ATCC 25922, and *Candida albicans* ATCC 10231) (Oxoid Corporation, England). The American Clinical and Laboratory Standards Institute (CLSI) standard was applied to evaluate outcomes. All specimens were inoculated in the appropriate culture medium and incubated at 35 °C in accordance with their respective requirements for 18 to 20 hours. The VITEK AMs60 compact automatic microbial analyzer (BioMerieux, France) was employed to identify the strains. A 30-g cefoxitin disk was used to detect methicillin-resistant staphylococci. Methicillin resistance was detected in coagulase-positive staphylococci when the inhibition zone diameter was ≤21 mm. Methicillin resistance was excluded in coagulase-negative staphylococci when the inhibition zone diameter was ≤24 mm.

### 2.4 Statistical analysis

WHONET software, version 5.5, and SPSS software, version 19.0 (SPSS, Inc., Chicago, IL), were used for statistical analysis. Categorical data are presented as frequencies and percentages.

## 3 Results

### 3.1 Annual distribution of pathogens in clinical samples

A total of 1891 strains of pathogens were detected from 3835 clinical specimens collected from 1449 burn patients over a 3-year period. The demographics of the 1449 patients were shown in Table 1.

**Table 1:**
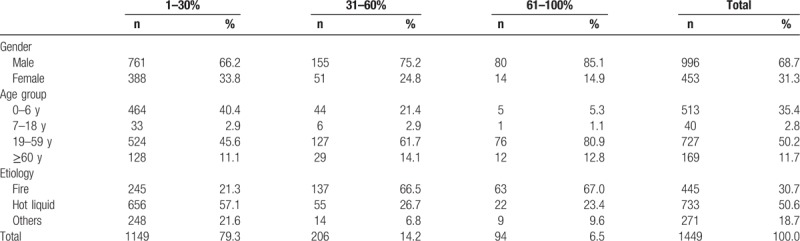
The demographics of 1449 patients.

The total detection rate was 49.3% (1891/3835). During these 3 years, the wound specimen strain detection rate showed a downward trend from 65.2% to 57.3%. The blood specimen strain detection rate was low with a range between 7.4% and 31.4% (Tables 2 and 3).

**Table 2:**
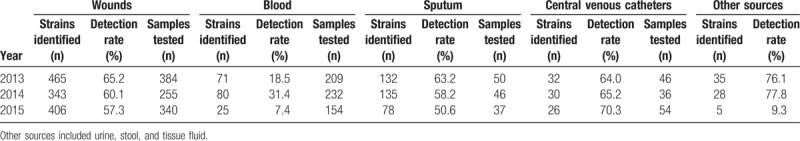
Annual detection rates of clinical samples from wounds, blood, sputum, central venous catheters, and other sources.

**Table 3:**
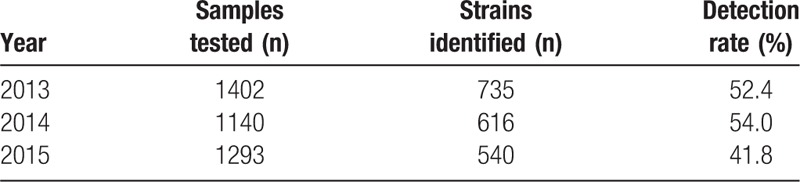
Annual detection rates of clinical samples from total samples.

### 3.2 Annual pathogen distributions and percentages

The most common pathogens isolated were gram-negative bacteria with 1089 strains (57.6%) followed by gram-positive bacteria with 689 strains (36.4%) and fungi with 113 strains (6.0%). The gram-negative bacteria detection rate was higher than that of gram-positive bacteria in every year. The 8 most common bacteria isolated were *S aureus* (19.0%), *Acinetobacter baumannii* (17.6%), *P aeruginosa* (16.7%), *Klebsiella pneumoniae* (7.4%), *Enterococcus faecalis* (4.5%), *Staphylococcus epidermidis* (3.6%), and *E coli* (3.2%). Over the study period, *A baumannii* and *P aeruginosa* exhibited a decreasing trend, while *S aureus* showed a rising trend (Table 4). Pathogen distributions and percentages among 1% to 30%, 31% to 60%, 61% to 100% TBSA patients were shown in Table 5. Clinical samples such as wound secretions, blood, sputum, and central venous catheters all exhibited a predominance of gram-negative bacteria. Sputum samples had the highest detection rate for fungi (Table 6).

**Table 4:**
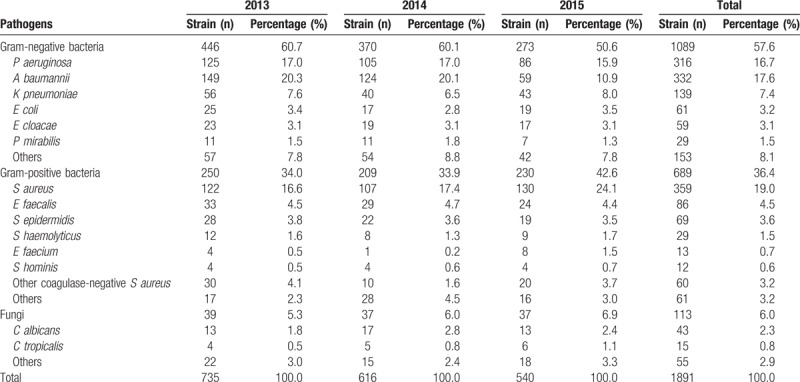
Annual pathogen distributions and percentages.

**Table 5:**
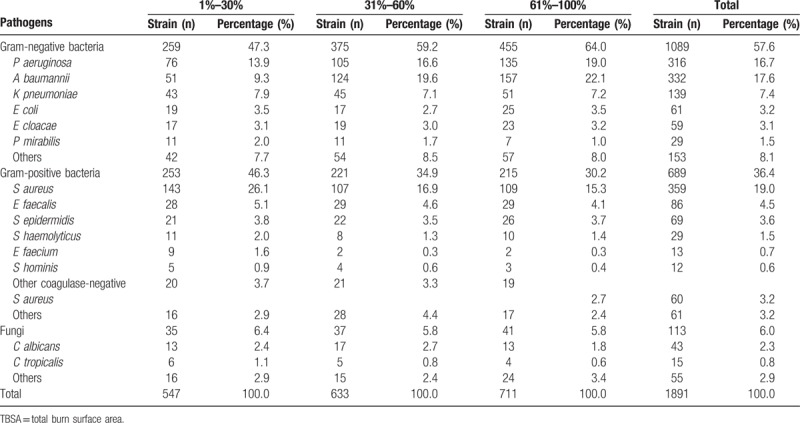
Pathogen distributions and percentages among 1% to 30%, 31% to 60%, 61% to 100% TBSA patients.

**Table 6:**
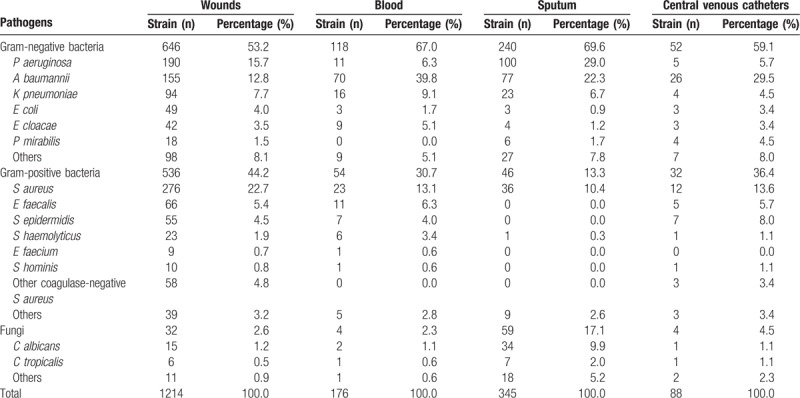
Pathogen distributions among different clinical samples.

### 3.3 The detection rate of methicillin-resistant staphylococci

Methicillin-resistant *Staphylococcus aureus* (MRSA) accounted for 74.1% (265/359) of *S aureus* isolates. The overall detection rate of MRSA was 14.0% with an increasing trend ranging between 12.2% and 16.7%. *S epidermidis* accounted for 40.6% (69/170) of coagulase-negative staphylococcal isolates, and 70.0% (48/69) of these were methicillin-resistant *Staphylococcus epidermidis* (MRSE). The overall detection rate of MRSE was 2.5% (48/1891), which changed little over the years (Table 7).

**Table 7:**
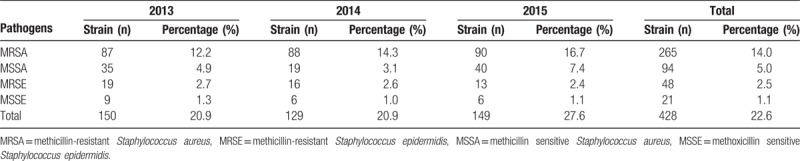
Annual detection rates of MRSA, MSSA, MRSE, and MSSE.

### 3.4 Resistance rates of gram-positive bacteria to antimicrobials

The resistance rates of the isolated bacteria to commonly used antimicrobials were investigated (Table 8). The resistance rates of MRSA to penicillin and ampicillin was 100%, whereas the resistance rates to erythromycin, clindamycin, gentamicin, tetracycline, and ciprofloxacin were >75%. In general, the high-resistance antibiotics were penicillin, ampicillin, and erythromycin, and the low-resistance antibiotics were teicoplanin, tigecycline, vancomycin, and linezolid. *E faecalis* was 100% resistant to quinupristin/dalfopristin synercid, whereas *Enterococcus faecium* was 100% sensitive to quinupristin/dalfopristin synercid. More than 50% of *E faecalis* was resistant to tetracycline, erythromycin, and rifampicin. Both enterococcal species were completely sensitive to vancomycin, tigecycline, and linezolid. Additionally, the frequency of *E faecalis* isolates was higher than that of *E faecium*. However, the drug resistance of *E faecalis* to penicillin, ciprofloxacin, levofloxacin, and ampicillin was lower than that of *E faecium*.

**Table 8:**
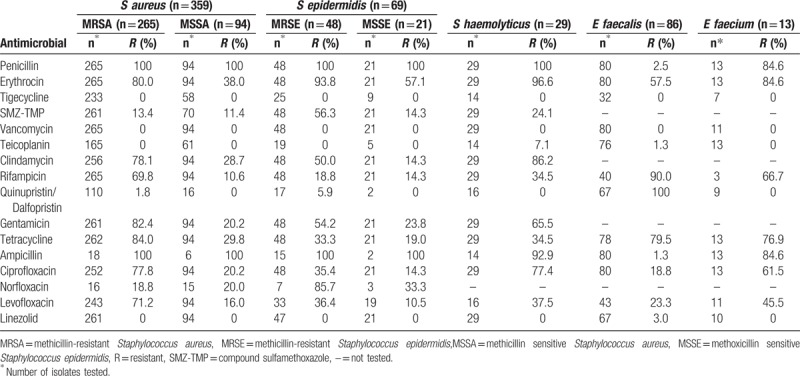
Resistance rates of gram-positive bacteria to antimicrobials.

### 3.5 Resistance rates of gram-negative bacteria to antimicrobials

The detection rates of extended-spectrum β-lactamases (ESBL) among *K pneumoniae* and *E coli* isolates were 44.6% (62/139) and 67.2% (41/61), respectively. For *A baumannii*, the most commonly isolated gram-negative bacterial strain, the resistance rate was 100% for ampicillin, amoxicillin, amoxicillin/clavulanate, and aztreonam, and >80% for third-generation cephalosporins such as cefotaxime, ceftazidime, ceftriaxone, and cefepime. The *A baumannii* resistance rates for carbapenems such as imipenem and meropenem were 58.5% and 83.3%, respectively.

In addition to resistance rates of *K pneumoniae* to ampicillin, amoxicillin, and cefazolin of 80%, these rates were 21% for meropenem and 15.4% for imipenem. More than 80% of *E coli* was resistant to ampicillin, piperacillin, cefazolin, amoxicillin, tetracycline, and sulfamethoxazole trimethoprim (Table 9).

**Table 9:**
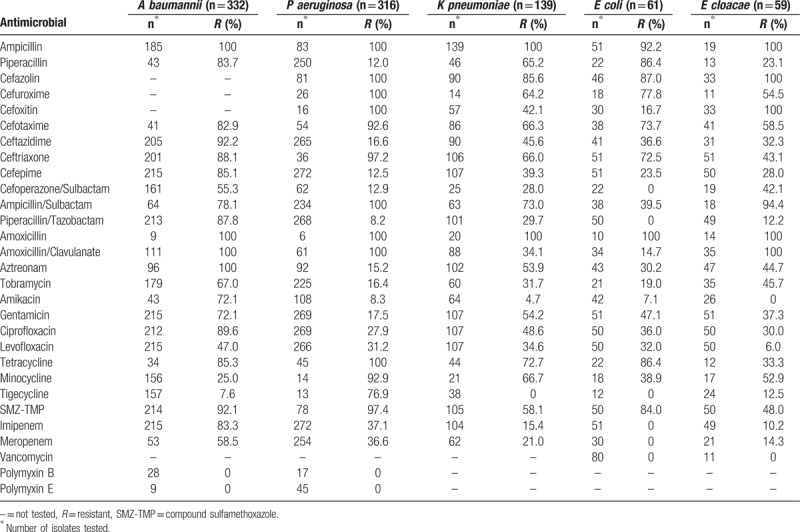
Resistance rates of gram-negative bacteria to antimicrobials.

### 3.6 Resistance rates of fungi to antimicrobial agents

The resistance rates of *C albicans* to amphotericin B, 5-fluorocytosine, and nystatin were 0%, and the resistance rate of *Candida tropicalis* to itraconazole was 50% (Table 10).

**Table 10:**
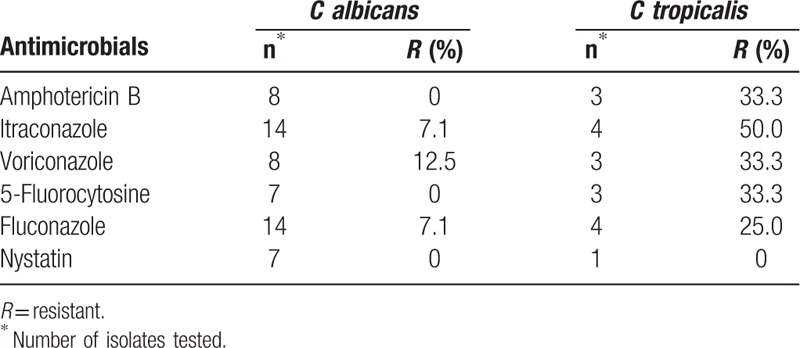
Resistance rates of fungi to antimicrobials.

## 4 Discussion

Nosocomial infections (NIs) are more likely to occur among burn patients due to the immunocompromising effects of burns, the nature of burns themselves, the intensive diagnostic and therapeutic procedures, and prolonged hospital stays.^[9]^ Infection rates are also associated with the burn wound degree, the need for surgery and age.^[10]^ The application of antibiotics remains an effective approach to control burn infections. The widespread use of broad-spectrum antibiotics, particularly cephalosporins and carbapenems, has been associated with the emergence of multiple-drug-resistant bacteria. Therefore, the monitoring of potential microbial infections among inpatients is critically important.

### 4.1 Pathogen detection rates and distribution characteristics

In total, 1891 strains of pathogenic bacteria or fungi were cultured from wound secretions, blood, central venous catheter, sputum, urine, stool, and tissue fluid. The overall detection rate was 49.3%. Specifically, the detection rates were 56.9% to 65.2% for wound secretions and 10.3% to 20.8% for blood, and these values are lower than the results obtained in the study conducted by Huang et al^[11]^ (64.7%–80.2% for wound secretions and 11.1%–51.5% for blood), who tested 571 pathogens from 1485 specimens. The differences in the detection rates likely resulted from the variations in the technology used for specimen collection and laboratory testing.

Among all the microbes detected, 36.4% (689/1891) were gram-positive bacteria, 57.6% (1089/1891) were gram-negative bacteria, and 6.0% (113/1891) were fungi. These results were similar to a study from Turkey,^[12]^ which was conducted retrospectively on a total of 250 microorganisms isolated from the burn-wound secretions of 179 patients between January 2009 and December 2011. However, gram-negative bacteria accounted for 64.4% (161/250) of the bacteria in that report, which was slightly higher than our findings.

Furthermore, our results showed that gram-positive bacteria increased and gram-negative bacteria decreased over these 3 years. This likely because of the clinical application of broad-spectrum antibiotics and the increased use of indwelling devices and other invasive procedures.

In our study, clinical samples such as wound secretions, blood, sputum, and central venous catheters all exhibited a predominance of gram-negative bacteria. This trend differed from an earlier report by Cen et al,^[13]^ who reported a predominance of gram-negative bacteria in wound secretions, whereas gram-positive bacteria predominated from blood cultures. Moreover, fungi were most prevalent in our sputum cultures, a finding that is also inconsistent with the results reported by Cen et al^[13]^ which indicated that fungi were most prevalent in urine cultures.

### 4.2 Analysis of bacterial resistance rates to antimicrobials

*S aureus* was the most prevalent bacterium observed. This result was similar to a previous study of 3615 microbial isolates from 114 patients with severe burns at the burn center of Shanghai Hospital (Shanghai, P.R. China) between 1998 and 2009, which found that *S aureus* accounted for 38.2% of all cases. In our study, MRSA represented 74.1% of *S aureus* isolates, approximating the result (73.0%) of another study in China by Wei et al^[14]^ (Gansu Provincial Hospital, 2008–2010). However, the majority of *S aureus* cases in our study were methicillin-resistant, a preponderance much higher than reported elsewhere, for example, by Dokter et al^[15]^ in New Zealand (0.4%), Fransén et al^[16]^ in Sweden (1.7%), Guggenheim et al^[5]^ in Switzerland (3%–16%), and Bayram et al^[12]^ in Turkey (19%). Generally, a relatively high percentage of *S aureus* isolates were MRSA in China. The resistance rates of MRSA to penicillin and ampicillin were 100% and 78.1% to 84% for other antibiotics such as erythromycin, tetracycline, and clindamycin. The resistance rates of MSSA and MSSE were relatively low to the aforementioned antibiotics except for penicillin and ampicillin. Vancomycin, teicoplanin, and tigecycline remained the most effective antibiotics against MRSA and MRSE with no strains resistant to these antibiotics. The policy named “search-and-destroy” has ensured a low prevalence of MRSA in health facilities and the population of the Netherlands,^[17]^ thereby establishing a valuable reference for China.

The coagulase-negative staphylococci detected included *S epidermidis* and *Staphylococcus saprophyticus*, both of which typically constitute part of the normal flora of the skin and mucous membranes.^[17]^ These microorganisms are generally associated with mucosal carriage as well as asymptomatic skin but are paradoxically recognized as some of the most frequent causative agents of device-associated infection (DAI) and hospital-associated infection (HAI).^[18–20]^ In our study, MRSE accounted for 70.0% (48/69) of *S epidermis* isolates, representing a relatively high detection rate. This finding should alert clinicians to control skin and mucosa disinfection strictly to avoid nosocomial infection.

The Enterococcus genus is among the normal flora of humans and animals, and ectopic microflora will lead to infection. Recently, Kozuszko et al^[21]^ suggested the presence of Enterococcus resistant to high concentrations of aminoglycosides; moreover, Faron et al^[22]^ reported vancomycin-resistant enterococci (VRE). In our study, the prevalence of VRE was 0%, which was much lower than the prevalence reported in Germany (11.2%), the United Kingdom (8.5%–12.5%), and Italy (9%).^[23]^

*P aeruginosa* was reported as a major pathogenic cause of infection after burn injuries in the United States.^[2]^ Previous studies have reported *P aeruginosa* as the most common isolate with high prevalence in regions such as the southwest of China^[10]^ (23.1%), Iran^[24]^ (26.7%), Iraqi Kurdistan^[25]^ (27%), Gaza^[26]^ (50%), and India^[27]^ (55%). The study by Ullah et al^[28]^ indicated that the incidence of *P aeruginosa* in burn wards was higher than in other wards. However, this finding was inconsistent with our observations. In our study, *P aeruginosa* was the second most prevalent gram-negative bacterium, accounting for 16.7% of bacterial isolates, which was slightly higher than in the studies by Coetzee et al^[29]^ (14.5%) and Bayram et al^[12]^ (12.0%). *P aeruginosa* was 100% resistant to ampicillin, ampicillin/sulbactam, amoxicillin, amoxicillin/clavulanic acid, cefuroxime, cefoxitin and cefotaxime, 97.5% resistant to sulfamethoxazole trimethoprim, and 8.2% to 12.9% resistant to cefepime, cefoperazone/sulbactam, piperacillin/sulbactam, and amikacin.

Recently, *A baumannii* has become one of the crucial causes of nosocomial infections.^[30]^ The occurrence of carbapenem-non-susceptible *A baumannii* (CNSAb) infections is becoming a growing problem in hospitalized burn patients.^[31]^*A baumannii* was the most common bacterium detected in most studies, for example, by Bayram et al^[12]^ in Turkey (23.6%), in Bahemia study^[32]^ of 341 severe burn patients admitted to an adult BICU from January 1, 2008 to December 31, 2012 in South Africa and in the Keen EF study of 3507 bacterial isolates from 460 BICU patients in a USA military burn center from January 2003 to December 2008.^[33]^ McDonald indicated that Acinetobacter spp. might be more prevalent in warm climates.^[34]^ This finding was consistent with our study because the Fujian province climate is quite warm, the province being located at a north latitude between 23°33′ and 28°20′ with an east longitude between 115°50′ and 120°40′ and temperature ranges over the 4 seasons of 11° to 27° in spring, 22° to 37° in summer, 19° to 37° in autumn, and 8° to 18° in winter. Our observations revealed *A baumannii* was the prominent pathogen among gram-negative bacteria, which presented high resistance to many antibiotics. The resistance rate to imipenem, the most effective broad-spectrum agent against gram-negative bacilli, was 83.3%. Moreover, diffusion disk testing using cefoperazone/sulbactam-containing inhibitors also showed a relatively high resistance rate of 55.3%. The resistance rate of *A baumannii* to ampicillin, piperacillin, aztreonam, amoxicillin, and amoxicillin/clavulanic acid was 100% and was >80% to cephalosporins.

The emergence of ESBL-producing strains among Enterobacteriaceae (*E coli*, *K pneumoniae*) has become a special concern.^[35]^ The detection rates of extended-spectrum β-lactamases among *K pneumoniae* and *E coli* were 44.6% (62/139) and 67.2% (41/61), respectively.

Fungal infections have been found to occur after the widespread use of antibiotic therapies, which have the effect of killing the beneficial bacteria that normally suppress fungi. More recently, Candida species have emerged as an important cause of invasive infections among patients in intensive care units.^[36]^ The most common fungi were *C albicans*, accounting for 38.1% (43/113) of fungal isolates. The antibiotic resistance of *C tropicalis* was higher than that of *C albicans*. A useful reference for China might be “Three steps to prevent invasive fungal diseases,” as concluded by Pemán and Salavert.^[37]^

### 4.3 Limitations

This was a multicentre study to investigate the epidemiology of bacteria among hospitalized burned patients. However, because of limited resources, we only investigated 1 province in the southeast of China. Because it was conducted retrospectively, we were unable to include the following steps: to test the homology of the MRSA; to analyze the impact of prehospital wound treatment on bacterial detection; to analyze the effect of antimicrobial administration on bacterial detection; and to analyze the effect of long-term catheterization on these bacteriological profiles. Future efforts are required to perform multicenter, prospective studies to develop dynamic monitoring for bacterial resistance.

## 5 Conclusions

Gram-negative bacteria represented the majority of pathogens detected. *S aureus*, *A baumannii*, *P aeruginosa*, *K pneumoniae*, and *E faecalis* were the 5 most common bacteria detected in the 4 study burn units. The resistance of MRSA and *A baumannii* to antibiotics was relatively high in Fujian province. High-resistance antimicrobials included penicillin, ampicillin, amoxicillin, cefazolin, and cefotaxime. Low-resistance antimicrobials included teicoplanin, tigecycline, vancomycin, and linezolid. Dynamic bacterial monitoring should be established to restrain the development of bacterial resistance.

## Acknowledgments

The authors thank Fujian Medical University Union Hospital, the 92nd Hospital of PLA, the 180th Hospital of PLA, and the First Hospital of Longyan City for their support.

## Author contributions

**Data curation:** Lin Li, Jia-xi Dai.

**Funding acquisition:** Lin Li, Le Xu.

**Investigation:** Lin Li, Jia-xi Dai, Zhao-hong Chen, Xiao-dong Chen.

**Methodology:** Lin Li, Zhao-hong Chen.

**Project administration:** Le Xu.

**Resources:** Zhao-hong Chen.

**Software:** Xiao-dong Chen.

**Supervision:** Xiao-yi Li, Yu-qing Wen, Min Liu.

**Validation:** Xiao-yi Li, Xiao-dong Chen.

**Writing–original draft:** Jia-xi Dai.

**Writing–review & editing:** Jia-xi Dai.
